# From Blurred Vision to Stage IV Lung Cancer: Choroidal Metastases

**DOI:** 10.7759/cureus.77835

**Published:** 2025-01-22

**Authors:** Shokri Siti-Sarah, Nurul Munirah Mohamad, Juanarita Jaafar, Ahmad Tajudin Liza-Sharmini

**Affiliations:** 1 Department of Ophthalmology and Visual Science, Universiti Sains Malaysia School of Medical Sciences, Kota Bharu, MYS; 2 Department of Ophthalmology, Hospital Sultanah Bahiyah, Alor Setar, MYS; 3 Department of Ophthalmology, Universiti Sains Malaysia School of Medical Sciences, Kota Bharu, MYS

**Keywords:** choroidal mass, choroidal metastasis, lung carcinoma, non-smoker lung carcinoma, overall well-being, women with choroidal metastasis

## Abstract

Metastatic choroidal tumors are the most common type of intraocular malignancy and are often linked to advanced-stage systemic cancers. Lung cancer, a leading cause of cancer-related mortality worldwide, occasionally presents with choroidal metastasis as the initial sign of systemic disease. This case series, which presents two distinct presentations of choroidal metastases as the first clinical manifestation of advanced lung adenocarcinoma, contributes to the understanding of the atypical presentations of lung cancer and the importance of considering ocular symptoms in the diagnosis of systemic diseases.

## Introduction

Metastatic choroidal tumors are the most prevalent form of intraocular malignancy [1]. Typically seen in patients with advanced-stage disease, the lung is the most common primary site of choroidal metastasis in men and the second most common in women, following breast cancer [1].

Visual changes or other ophthalmic symptoms as the initial manifestation of lung cancer are a rare but significant occurrence. Advances in oncology and diagnostic imaging have led to increased survival rates and earlier detection of metastatic lesions, contributing to a rising incidence of choroidal metastases [2]. Lung and breast cancers account for the majority of cases, with lung cancer responsible for 20-29% of ocular metastases [2].

This report presents two cases of women with non-small cell lung cancer (NSCLC) with choroidal metastases as the first sign of metastatic disease.

## Case presentation

Case 1

**Figure 1:**
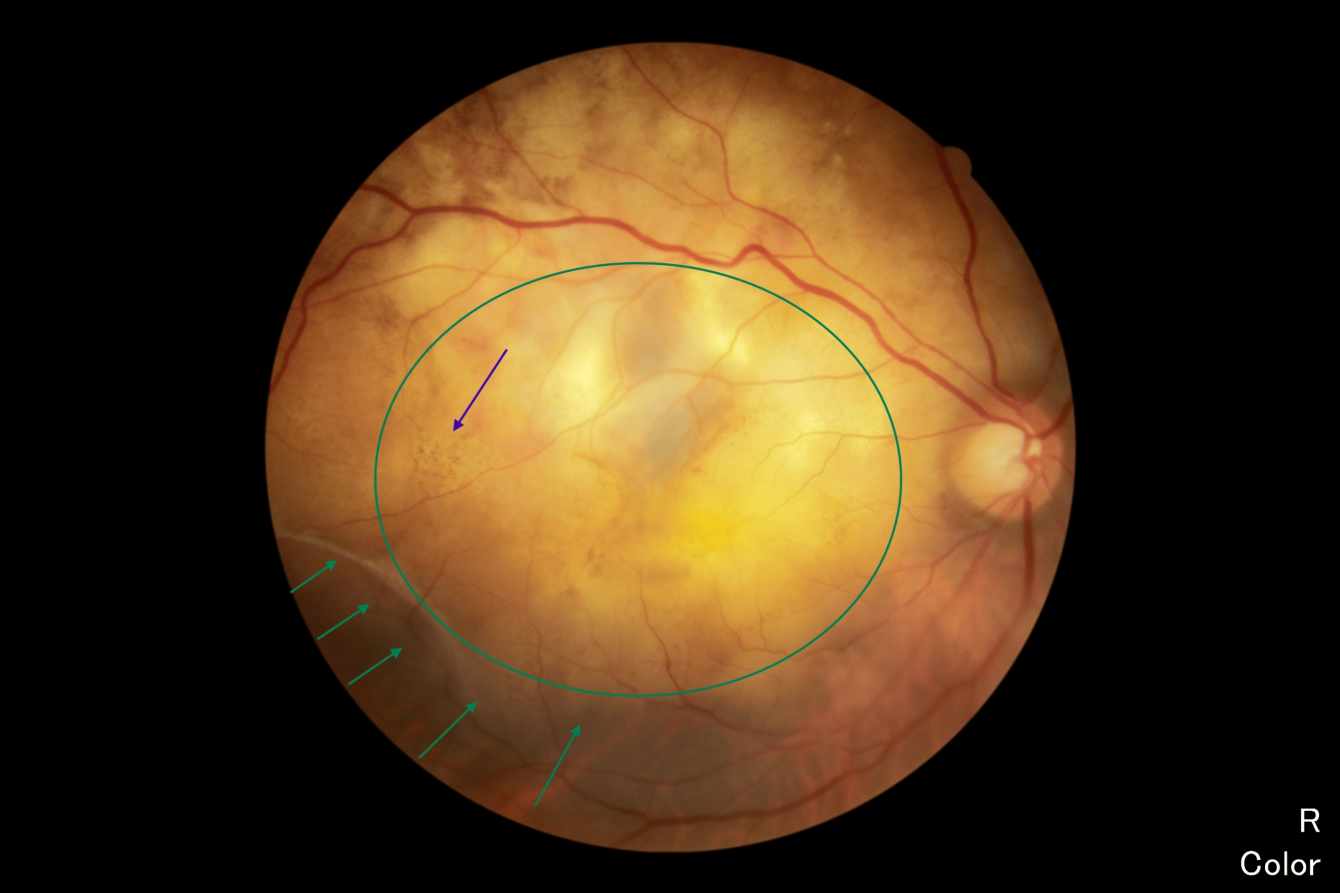
Funduscopy Right fundoscopy showed an irregular lobulated mass involving the whole macula extending to the temporal retina and superotemporal arcade (green circle) with overlying retina hyperpigmentation (blue arrow). Serous retinal detachment near the lesion (green arrow).

**Figure 2:**
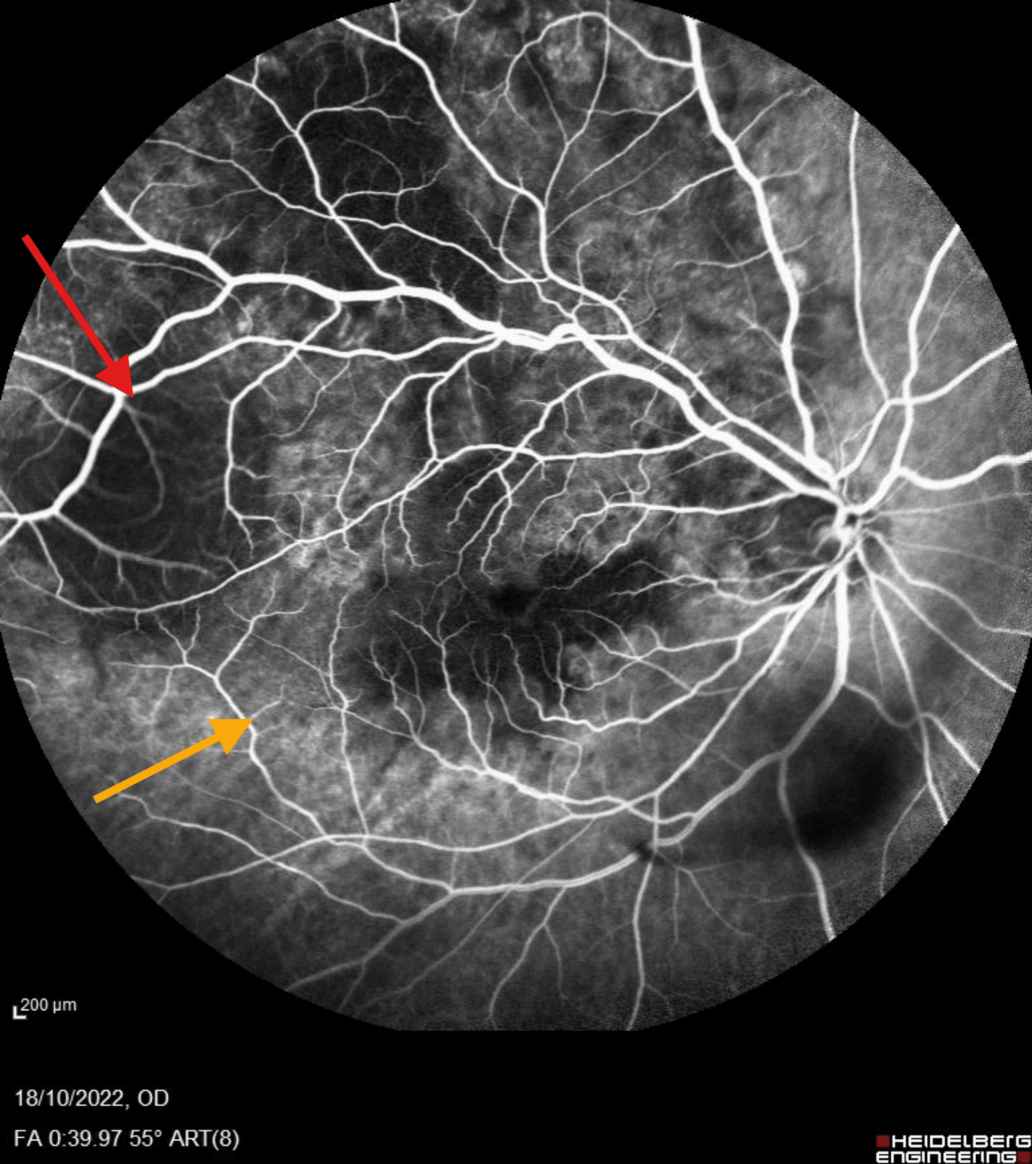
FFA FFA demonstrated a masking effect in the posterior pole and superotemporal arcade without evidence of dual circulation (red arrow). Pooling of fluorescein dye is seen, indicating subretinal fluid or leakage from the tumor (yellow arrow). FFA, fluorescein angiography

**Figure 3:**
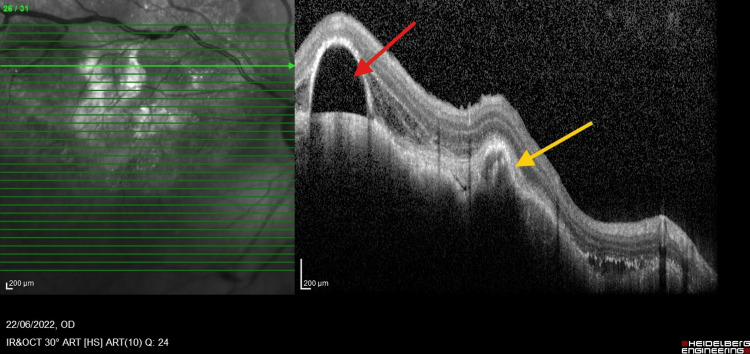
OCT OCT revealed irregularities in the choroidal structure, with a hyporeflective area indicating subretinal fluid (red arrow) and pigment epithelial detachment. Hyperreflectivity at the RPE (yellow arrow) may indicate an infiltrative tumor or subretinal fibrosis. OCT, optical coherence tomography; RPE, retinal pigment epithelium

A 65-year-old female, a retired government pensioner with a history of dyslipidemia, presented with a six-month progression of central scotoma in her right eye. Her family history was notable for malignancies, including gynecological cancer in her mother and lung cancer in her sister. The presence of a family history of cancer may have influenced the suspicion of systemic malignancy and the decision to conduct further investigations. On examination, her best-corrected visual acuity (BCVA) was 6/36 in the right eye (OD) and 6/9 in the left eye (OS). The bilateral anterior segment and intraocular pressure were normal. Fundoscopy of the right eye revealed an irregular, lobulated mass involving the macula, extending to the temporal retina and superotemporal arcade (Figure 1). Further evaluation with fluorescein angiography (FFA) demonstrated a masking effect in the posterior pole and superotemporal arcade without evidence of dual circulation (Figure 2). Optical coherence tomography (OCT) revealed irregularities in the choroidal structure, subretinal fluid, and pigment epithelial detachment (Figure 3). Systemically, the patient reported occasional shortness of breath on exertion and only noticed weight loss after six months of experiencing blurred vision.

**Figure 4:**
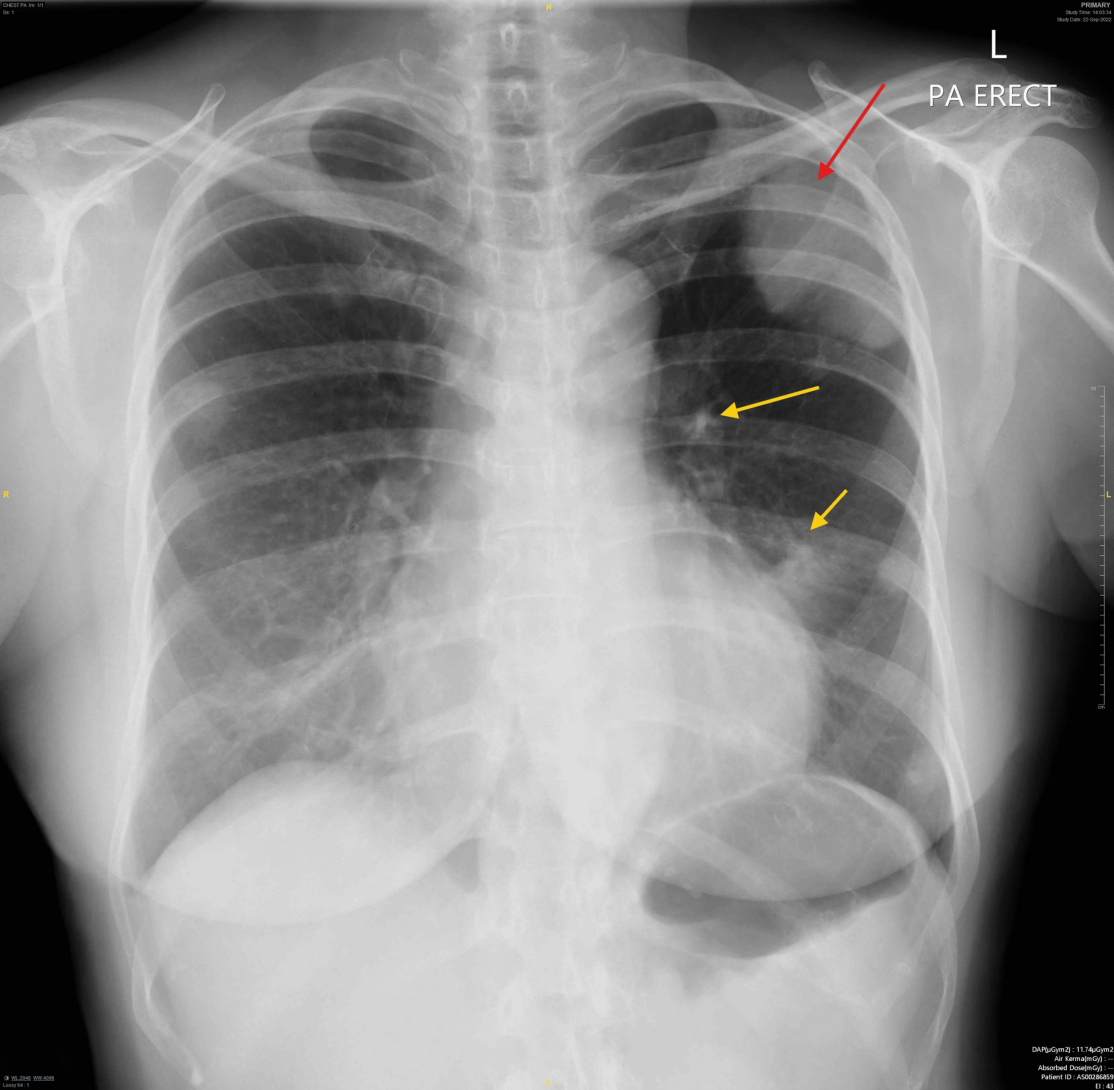
Chest X-ray Posterior-anterior chest X-ray revealed heterogeneous opacity in the left upper zone, which could represent a mass lesion (red arrow) with lung nodules (yellow arrow).

Due to the ocular findings, a systemic malignancy was suspected, which led to further investigations. A chest X-ray revealed a left pleural-based mass with bilateral lung nodules (Figure 4). Computed tomography (CT) of the lung confirmed the presence of a left upper lobe mass with pleural involvement, multiple pulmonary nodules, and enlarged regional lymph nodes. Endobronchial ultrasound-guided biopsy confirmed the diagnosis of lung adenocarcinoma, with genetic testing revealing an epidermal growth factor receptor (EGFR) exon 19 deletion mutation. Blood investigations, including tumor markers, renal function tests, liver function tests, and a complete blood count, were unremarkable. The final diagnosis was stage IV lung adenocarcinoma (T3 N1 M1a) with ocular metastasis. Blood investigations, including tumor markers, renal function tests, liver function tests, and a complete blood count, were unremarkable.

The patient was initiated on afatinib 20 mg daily, a tyrosine kinase inhibitor (TKI) targeting EGFR-mutated lung cancers. After two months, the dose was increased to 30 mg daily. Adverse effects included minimal facial acne and dry skin. A follow-up CT thorax after six months demonstrated a reduction in the size of the left lung mass and smaller mediastinal and left perihilar nodes. However, despite systemic response, her right eye vision deteriorated to hand movements (HM).

After two years on afatinib, disease progression was noted, evidenced by an increase in the size of the left upper lobe mass and bilateral lung nodules. The patient received three cycles of carboplatin/pemetrexed chemotherapy, followed by osimertinib 80 mg daily. Four months after initiating osimertinib, CT imaging showed a stable left upper lobe mass with new right middle lobe consolidation. However, right eye vision remained HM despite a reduction in the size of the choroidal mass.

Case 2

**Figure 5:**
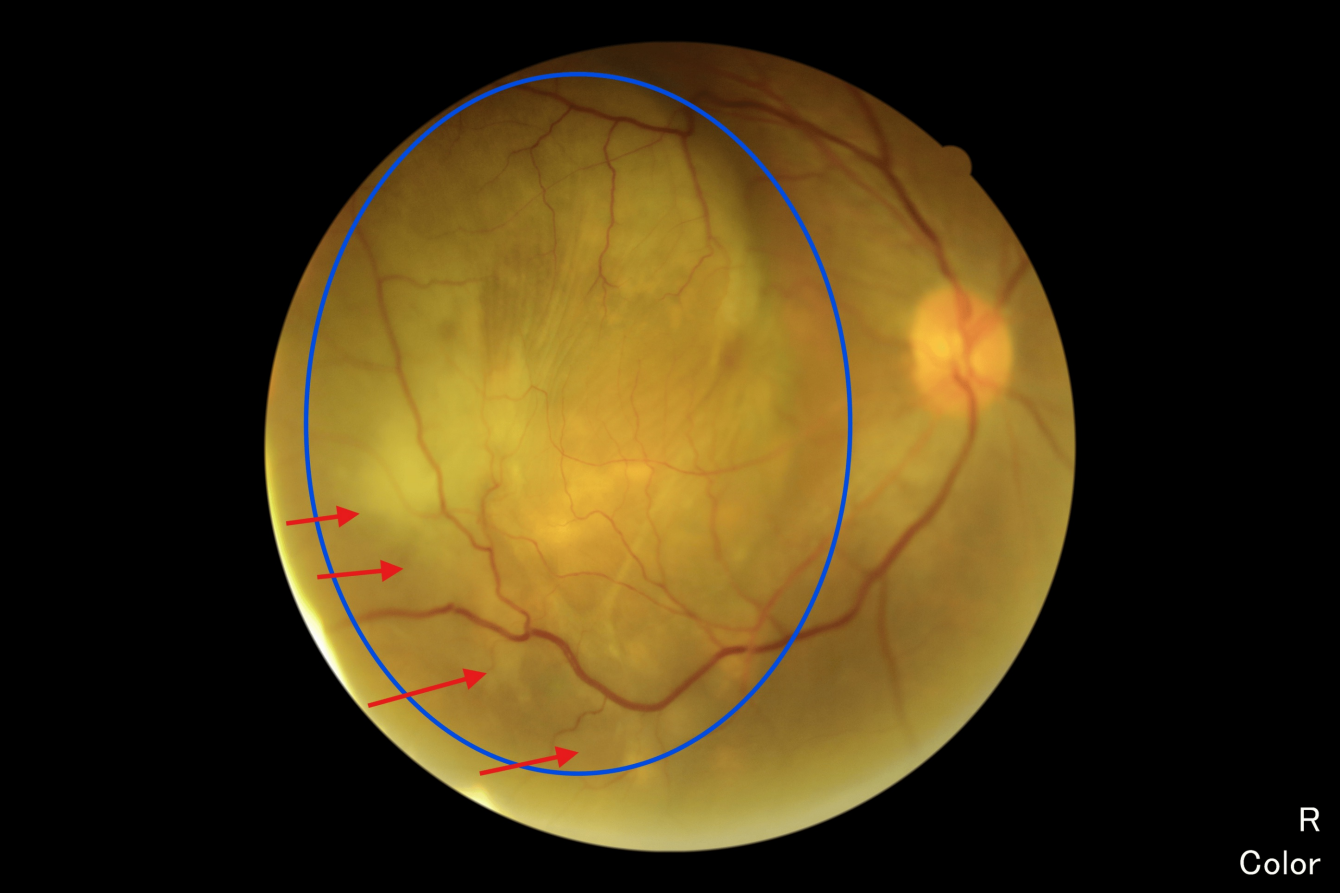
Funduscopy Funduscopy showed a yellowish, elevated lesion with diffuse, irregular borders at the posterior pole (blue circle). The surrounding retina seems to have areas of serous retinal detachment (red arrow).

**Figure 6:**
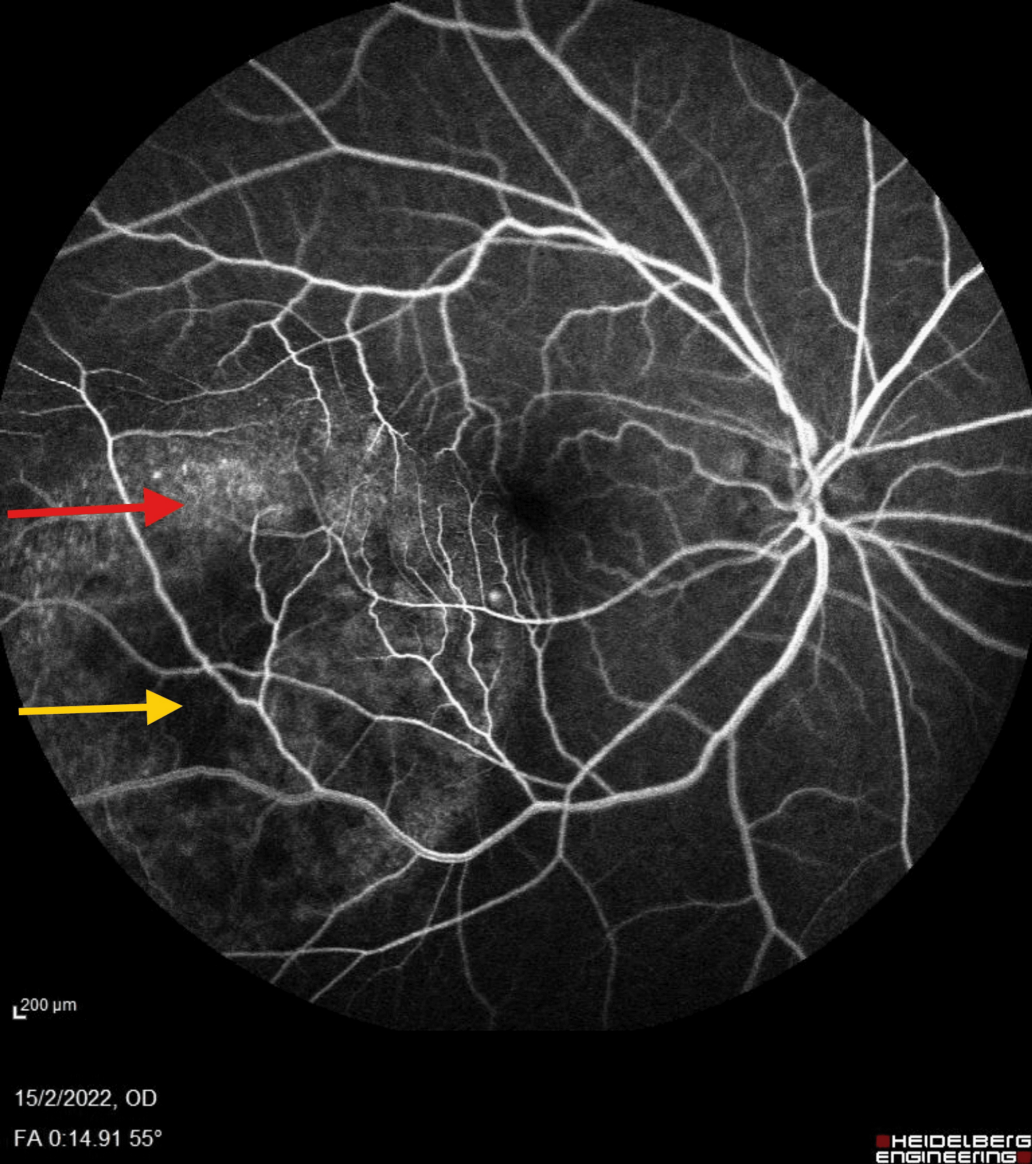
FFA Areas of serous retinal detachment appeared hyperfluorescent due to the pooling of dye in the subretinal space (red arrow). Hypofluorescence due to blockage of choroidal fluorescence by the overlying RPE changes and tumor mass (yellow arrow). FFA, fluorescein angiography; RPE, retinal pigment epithelium

**Figure 7:**
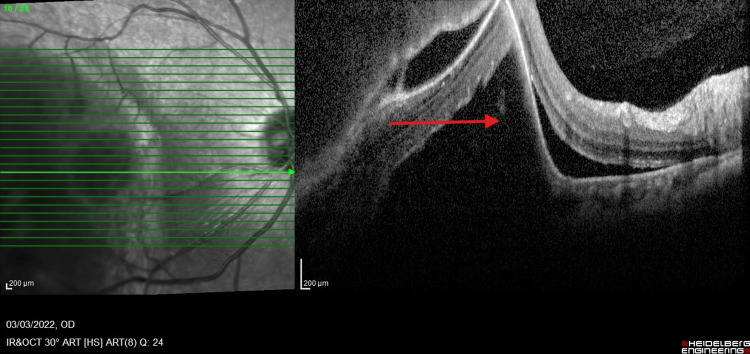
OCT OCT scan demonstrates a dome-shaped elevation of the choroid, which corresponds to the metastatic lesion seen in the fundus photograph (red arrow). This elevation displaces the overlying retina. OCT, optical coherence tomography

A 51-year-old female teacher presented with a two-month history of paracentral nasal scotoma in her right eye. She reported no significant systemic complaints but described profound emotional distress following the recent loss of her only child to brain cancer. On examination, her BCVA was 6/18 in the right eye and 6/9 in the left eye. Fundoscopy of the right eye revealed a yellowish, solid elevation located inferonasal to the fovea, along with hypopigmented central macular changes (Figure 5). FFA excluded the possibility of choroidal melanoma (Figure 6). OCT demonstrates a dome-shaped elevation of the choroid, which corresponds to the metastatic lesion (Figure 7). B-scan ultrasonography showed a double-hump hyperechoic lesion lacking choroidal melanoma's classic features.

**Figure 8:**
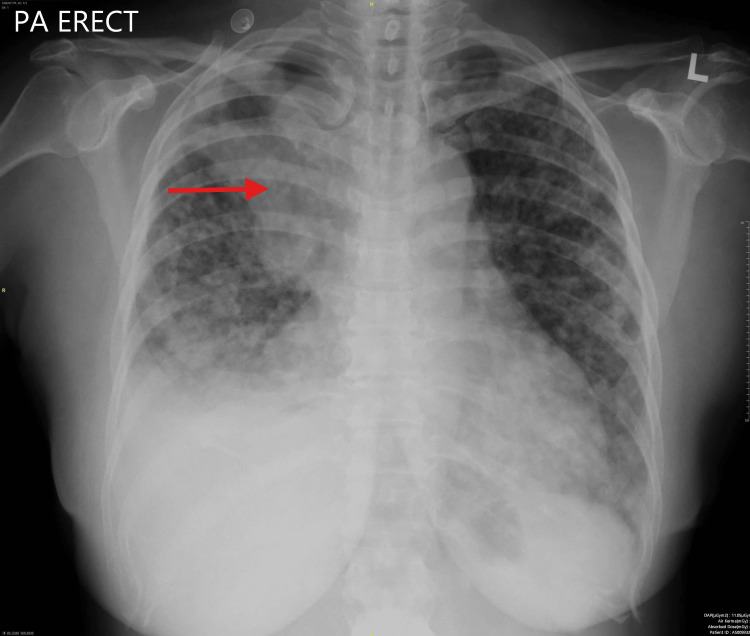
Chest x-ray A chest radiograph revealed mediastinal widening with a mass in the right upper lobe (red arrow).

Further systemic investigations were conducted. A chest radiograph revealed mediastinal widening with a mass in the right upper lobe (Figure 8). Magnetic resonance imaging (MRI) of the brain and orbits confirmed a lesion within the right globe. CT scans of the thorax, abdomen, and pelvis demonstrated an enhancing right lung mass, multiple pulmonary nodules, and metastases to the mediastinum, liver, and bone. A CT-guided biopsy confirmed stage 4 adenocarcinoma of the lung with widespread metastases. Molecular testing revealed the absence of EGFR mutations.

Based on these findings, a diagnosis of ocular metastasis secondary to lung adenocarcinoma was made. The patient declined palliative chemotherapy and opted for supportive care. Over the following months, her vision in the right eye deteriorated to counting fingers. This decline was further complicated by the development of phacomorphic glaucoma, which ultimately led to blindness in the affected eye despite efforts to control intraocular pressure.

## Discussion

Intraocular metastasis is recognized as the most common malignancy affecting the eye [1]. The highly vascular uveal tract is the primary site involved in ocular metastasis, with the choroid (88%) being the most frequently affected region, followed by the iris (9%) and the ciliary body (2%) [3]. Due to its rich vascular supply, the choroid is a common site for disseminating lung cancer cells [4].

In female patients, choroidal metastases most commonly originate from breast, lung, or gastrointestinal cancers, as well as malignant melanoma. In male patients, the primary sources are typically lung, gastrointestinal, pancreatic, prostate, or kidney cancers [3]. According to Kreusel KH's study, among patients presenting with choroidal metastasis as an initial symptom, 58% had lung cancer, and 28% had breast cancer [4]. Diagnosis of choroidal metastasis often coincides with systemic disease progression. However, in 44% of lung cancer patients, it represents the first clinical indication of metastatic spread [3].

The diagnosis of choroidal metastases is primarily based on clinical findings supported by imaging techniques, including ultrasound, FFA, orbital CT scans, OCT, nuclear MRI, and, in some cases, puncture-aspiration biopsy. Choroidal metastases in advanced-stage lung cancer indicate widespread dissemination, which is almost certain. Biopsy of a choroidal lesion is generally performed only when the lesion is isolated and unique, and no primary neoplasia is identified [5].

A multidisciplinary approach integrating local and systemic therapies is crucial for effective management, even though the overall prognosis remains unfavorable. Survival rates for patients with choroidal metastases are 57% at one year and drop to 23% at five years. Particularly concerning is that patients with lung cancer face a significantly reduced five-year survival rate of just 13% [6]. Remarkably, our patient has survived choroidal metastasis of lung adenocarcinoma for 24 months. The management of intraocular metastases largely depends on the patient's overall clinical status and is primarily palliative [1,7].

The presence of choroidal metastases suggests hematogenous cancer spread, and treatment aims to improve quality of life while restoring or preserving vision. Available treatment options include enucleation, exenteration, transpupillary thermotherapy, photocoagulation, photodynamic therapy, chemotherapy, and orbital irradiation [1,7].

In patients with chemo-responsive primary tumors, chemotherapy alone may suffice. No additional ocular treatment is typically indicated in asymptomatic patients already undergoing systemic chemotherapy. In challenging cases that do not respond to standard treatments, radiation therapy often emerges as a valuable option [7].

## Conclusions

Choroidal metastasis is a crucial early sign of metastatic lung cancer, highlighting the need for awareness and collaborative care among healthcare professionals. These cases clearly illustrate the pivotal role that ophthalmologists have in identifying systemic malignancies and the significant opportunities for improving the management of both ocular and systemic conditions concurrently. Recognizing choroidal metastasis as an early sign of metastatic lung cancer requires assertive awareness and well-coordinated management strategies. These instances firmly establish the crucial contributions of ophthalmologists in diagnosing systemic cancers and addressing the challenges of simultaneously treating both ocular and systemic diseases effectively.

## References

[1] Arepalli S, Kaliki S, Shields CL (2015). Choroidal metastases: origin, features, and therapy. Indian J Ophthalmol.

[2] Mathis T, Jardel P, Loria O (2019). New concepts in the diagnosis and management of choroidal metastases. Prog Retin Eye Res.

[3] Shields CL, Shields JA, Gross NE, Schwartz GP, Lally SE (1997). Survey of 520 eyes with uveal metastases. Ophthalmol.

[4] Kreusel KM, Wiegel T, Stange M, Bornfeld N, Hinkelbein W, Foerster MH (2002). Choroidal metastasis in disseminated lung cancer: frequency and risk factors. Am J Ophthalmol.

[5] Jakobiec FA, Ramsey DJ, Stagner AM, Wu DM, Yoon MK (2015). Pulmonary adenocarcinoma metastatic to the choroid diagnosed by biopsy of an extrascleral nodule. Ocul Oncol Pathol.

[6] Shields CL, Welch RJ, Malik K (2018). Uveal metastasis: Clinical features and survival outcome of 2214 tumors in 1111 patients based on primary tumor origin. Middle East Afr J Ophthalmol.

[7] Kanthan GL, Jayamohan J, Yip D, Conway RM (2007). Management of metastatic carcinoma of the uveal tract: an evidence-based analysis. Clin Exp Ophthalmol.

